# Response to Morita et al., Re: *Continuous Deep Sedation* (DOI: 10.1089/pmr.2021.0058)

**DOI:** 10.1089/pmr.2022.0018

**Published:** 2022-06-07

**Authors:** Bert Broeckaert

**Affiliations:** Department of Theology and Religious Studies, KU Leuven Humanities and Social Sciences Group, Leuven, Belgium.

As an ethicist involved in palliative care research for many years, I want to respond to the thought-provoking article by Morita and colleagues.^1^ In 2000, I introduced a new definition of sedation (“the intentional administration of sedative drugs in dosages and combinations required to reduce the consciousness of a terminal patient as much as necessary to adequately relieve one or more refractory symptoms”), a threefold distinction (intention/act/result) between sedation and euthanasia and a new term, palliative sedation, that would convey this central message that sedation is all about symptom control and thus proportionality is its key ingredient.^2–4^ From this perspective, that I developed in many articles on different aspects of palliative sedation since, I want to respond to the article of Morita et al.

1. Instead of replacing the concept of intention by the (very similar) concept of treatment goal, I prefer to shift the focus from the intention (or goal) itself to the *act* that materializes this intention. If one wants to translate the intention to relieve symptoms into a fitting action, one automatically ends up with the concept of proportionality: giving as much medication and lowering consciousness *as much as necessary*.
2. Because of this shared intention and proportionality, there are no rigid boundaries between the different subtypes of palliative sedation; intermittent and light forms of sedation often evolve into continuous and deep sedation. Palliative sedation is essentially *dynamic*.
3. Proportionality does not imply that one should always start with the lightest form of sedation. It simply means choosing the grade or form of sedation that best responds to the refractory symptoms encountered. Indeed, in some cases one could and should immediately opt for deep and continuous sedation. However, what I continue to oppose is the creation of a *separate* category “continuous deep sedation until death.” By doing this, one loses sight of the essential dynamic quality and proportional nature of palliative sedation and one quickly runs the risk of getting bogged down in euthanasia-like practices.
4. It is simply wrong to assume that one could or should base one's definition of sedation on empirical studies. I am not only thinking about Moore's naturalistic fallacy (“one can never derive an *ought* from an *is*”) here, but more specifically also about the fact that several of the empirical studies mentioned are including so-called cases of palliative sedation performed by physicians without sufficient expertise and physicians who are even using sedation with the explicit (co-)intention of hastening death (17% in one study!). Do these findings really force us to change our definition or understanding of palliative sedation and to recognize and name slow euthanasia too as a type of (palliative) sedation?
